# How the COVID-19 pandemic changed stakeholder engagement processes in sustainability research in the long-term

**DOI:** 10.12688/f1000research.145238.1

**Published:** 2024-05-07

**Authors:** Diana Süsser, Amanda Schibline, Andrzej Ceglarz, Johan Lilliestam, Vassilis Stavrakas, Pia-Johanna Schweizer

**Affiliations:** 1 Institute for European Energy and Climate Policy, Amsterdam, 1043GR, The Netherlands; 2 Renewables Grid Initiative, Berlin, 12101, Germany; 3 Technical University Munich, Munich, 80333, Germany; 4 Sustainability Transition Policy, Friedrich Alexander University Erlangen-Nürnberg, Nürnberg, 90403, Germany; 5 Τechnoeconomics of Energy Systems laboratory (TEESlab), Department of Industrial Management and Technology, University of Piraeus, Piraeus, 18534, Greece; 6 Research Institute for Sustainability, Helmholtz Centre Potsdam, Potsdam, 14467, Germany

**Keywords:** COVID-19, stakeholder engagement, sustainability research, EU

## Abstract

**Background:**

The COVID-19 pandemic affected stakeholder engagement in sustainability research projects in many ways. But which effects appear permanent today, after the pandemic ended?

**Methods:**

To address this, we interviewed researchers and stakeholders and carried out a survey among European sustainability research projects in 2022.

**Results:**

We find that the pandemic years disrupted stakeholder-based research, also with lasting effects. The forced shift to online modes showed how digital engagement can bring benefits in terms of easier and more efficient stakeholder engagement, but also that important aspects are lost, particularly regarding intensity of collaboration and depth of insights. Whether to go online or stay offline depends largely on the research objective, which stakeholders to involve, and how well researchers and stakeholders already know each other. Most researchers and stakeholders want to continue online collaboration in the long term, especially those with positive online collaboration experiences from the pandemic years.

**Conclusions:**

The pandemic has a long-term impact on stakeholder engagement in research; online engagement cannot replace all benefit of previous in-person interactions with stakeholders, but it has led to digital innovations and expanded the engagement portfolio. Our research has provided qualitative insights into the impact of the pandemic on stakeholder engagement in various sustainability research projects and the implications for the long-term future that are relevant to researchers and funding agencies.

## Introduction

Solving sustainability problems such as the energy transition requires scientific and non-scientific understandings and a broad range of knowledge ( Lawrence et al. 2022). In many research projects, such knowledge is generated together with different actors representing society. Stakeholders range from (but are not limited to) citizens to representatives of businesses and non-governmental organisations to policymakers at local and international levels, who are either affected by the research or have a particular interest in the research outcomes. Effectively engaging stakeholders in research is never an easy task due to limited availability and differing priorities, but the COVID-19 pandemic ^1^ has added a new complexity to it.

The containment measures introduced after the outbreak of the pandemic in early spring of 2020 have impacted sustainability transitions, such as in the energy sector ( Quitzow et al. 2021), and caused changes in consumptions patterns ( Cohen 2020). But the pandemic has also disputed the ways how research has been carried out across different disciplines ( Corbera et al. 2020; Köpsel et al. 2021; Leal Filho et al. 2020; Schwarz et al. 2020). The new COVID-19 reality has particularly influenced the way “how qualitative research is conducted on topics related to sustainability science” ( Santana et al. 2021:1061). Stakeholder engagement is important to many sustainability research projects ( Fazey et al. 2020; Lutz & Bergmann 2018; Mielke et al. 2016), which was particularly affected by the social distancing measures imposed ( Jäger et al. 2023; Köpsel et al. 2021; Süsser et al. 2021; Tobin et al. 2020). In consequence, many activities have been cancelled, delayed, or moved online ( Köpsel et al. 2021; Süsser et al. 2021).

Alternations to the stakeholder engagement required appropriate preparations and considerations of different dimensions, such as: (1) technology (which online tools are available to allow the stakeholders to be actively involved); (2) modifications to methodology and agenda (elements to be adjusted depending on the features of the technology applied); (3) scheduling (in terms of a reduced time effort, but also considering possible disturbances, such as presence of children at home during working hours) ( Tobin et al. 2020). However, online formats led to a reduced satisfaction of the involved stakeholders and potential exclusion of social groups ( Beaunoyer et al. 2020; Köpsel et al. 2021; Santana et al. 2021; Süsser et al. 2021). The pandemic brought not only physical challenges related to social distancing measures, but also additional psychological and ethical challenges related to new stress factors caused by COVID-19-related uncertainties ( Santana et al. 2021).

On the other hand, the COVID-19 pandemic can be thought of as a disrupter of current modes of doing stakeholder research, comprising opportunities for new and different forms of involving stakeholders in research ( Jäger et al. 2023; Tobin et al. 2020). For example, specific methodologies as well as different engagement tools and means (such as presentations, posters, group discussions, surveys, and feasibility tools) could be adapted accordingly to fit into the online workshop formats ( Jäger et al. 2023; Tobin et al. 2020). Furthermore, online engagement can help to reduce the resource use, including the involvement time ( Jäger et al. 2023; Süsser et al. 2021; Tobin et al. 2020) and lead to additional, positive side effects, such as a stronger involvement of the entire research team ( Jäger et al. 2023). Thus, the pandemic provided opportunities for communicating and facilitating sustainability transitions within and beyond the research sphere ( Bodenheimer & Leidenberger 2020).

While previous research has examined the short-term impacts, it is not yet certain to what extent COVID-19 triggered long-term changes in stakeholder-based research, or whether research will return to its previous business-as-usual. With this article, we follow up on our own research ( Süsser et al. 2021) and that of other researchers on the impact of the COVID-19 pandemic on stakeholder engagement ( Jäger et al. 2023; Köpsel et al. 2021) by examining the medium-term impact of the pandemic on sustainability research projects in the European Union (EU) and the long-term prospects for stakeholder engagement. Our main research question is: How has the COVID-19 pandemic changed stakeholder engagement processes in sustainability research in medium and long-term? First, we investigate what type of stakeholders were involved in research projects and why. Second, we examine how researchers and stakeholders retrospectively evaluate the research process carried out online and its impacts on the outcomes. Third, we examine when and why it is better to take activities online or run them as in-person events. Fourth, we explore the main perspectives from the COVID-19 ‘disruption’ on how researchers want to engage stakeholders and how stakeholders want to be engaged in future sustainability research projects.

To answer these questions, we conducted 15 interviews with researchers and stakeholders from 11 European sustainability research projects to gain a better understanding of the changes brought about by the pandemic as well as the viewpoints associated with the changes and the reasons behind them. Based on the questionnaire from our 2020 survey ( Süsser et al. 2021) and the interviews of this study, we re-designed an online survey and distributed it to a larger sample of European research projects with stakeholder engagement to reveal the different impacts of COVID-19 and draw conclusions about long-term impacts of the crisis.

## Methods

### Interviews

From April to October 2022, we conducted 15 interviews with 13 researchers (“R”) and 2 stakeholders (“S”) to understand how they have experienced the impact of the pandemic on the research process and outcomes, and how they would decide about future online and offline engagement. We interviewed different sustainability research projects: three on agriculture and food, two on energy, two on climate, two on marine management, one on water management, and one on material risk management. Nine projects were funded by the EU Horizon 2020 research programme, whereas the remaining two projects had other national funders, such as ministries and foundations. The authors of this article have not been involved in any of these projects. We selected the projects from which we recruited our interviewees based on three criteria: (i) a topical diversity of projects covering different sustainability topics; (ii) stakeholder engagement was an integral part of the projects; (iii) projects starting no later than January 2020 and running at least until the end of 2020. Projects that fulfil these criteria have been selected from the CORDIS data base and been contacted with an interview request. In addition, we screened for projects among the participants of two thematically related surveys in 2020 ( Köpsel et al. 2021; Süsser et al. 2021)where participants indicated a high importance of stakeholder engagement and provided us with the contact details; researchers and/or coordinators from four projects agreed to be interviewed.

### Survey

**Figure 1.**
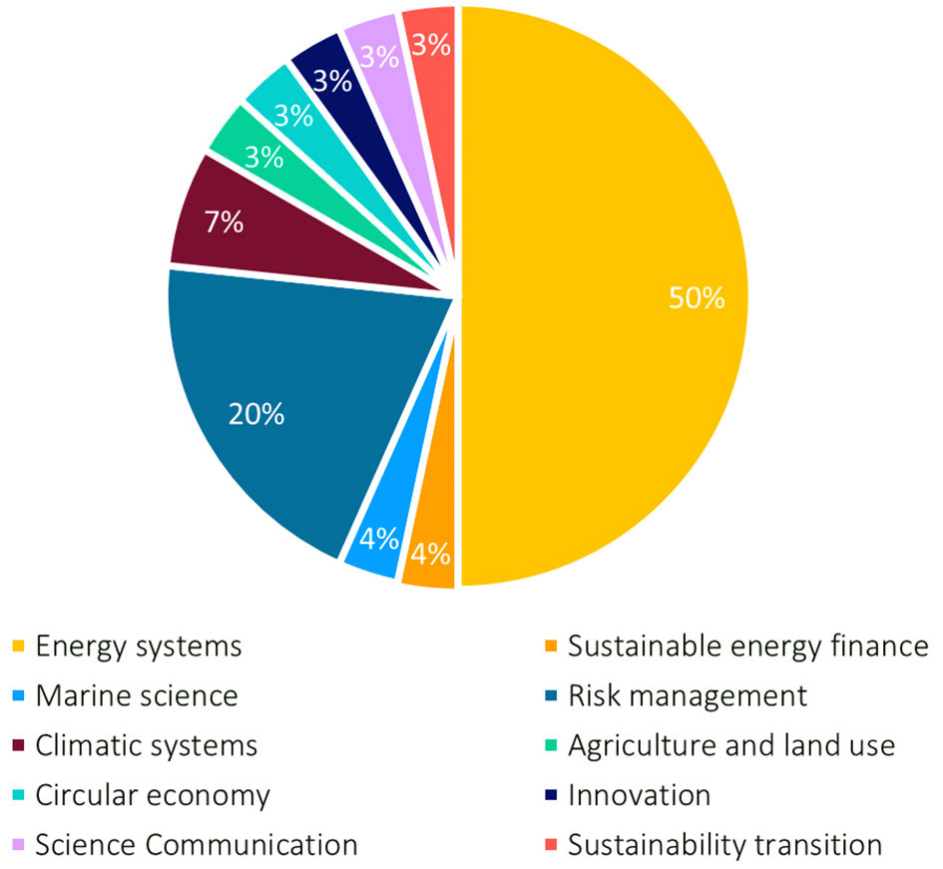
Research fields of participating researchers in the survey, n = 30.

In autumn 2022, we conducted an online survey to explore the longer-term impacts of the pandemic on research projects and perspectives for future projects. We contacted the same projects from energy research and marine sciences that participated in our surveys in 2020 and invited other sustainability researchers to complete the survey via existing networks (incl. social media) and other project collaborations. We received responses from 26 different projects with responses from up to four researchers from a single project. This is a much lower response in comparison to the survey we conducted in 2020. Similarly, more than twice as many respondents as in 2020 started the survey but did not finish it; this can have different reasons such as that it was too long, or respondents faced technical issues. In our analysis we included only 30 completed responses (“survey_ID#”) for qualitative quotes ( Figure 1). The responses came from 26 projects representing different fields; half of them from energy research, sixty-eight percent of the respondents were Horizon 2020 projects. Most projects (14) started in 2019, and end(ed) in 2023 (16 projects). The survey’s respondents had a diverse work experience in their field and various levels of experience regarding stakeholder engagement; many (about 40%) had 1-5 years of experience. Two-thirds of the respondents were men, a bit less than a third women and some preferred not to indicate their sex.

## Results

### Stakeholder engagement in sustainability research

**Figure 2.**
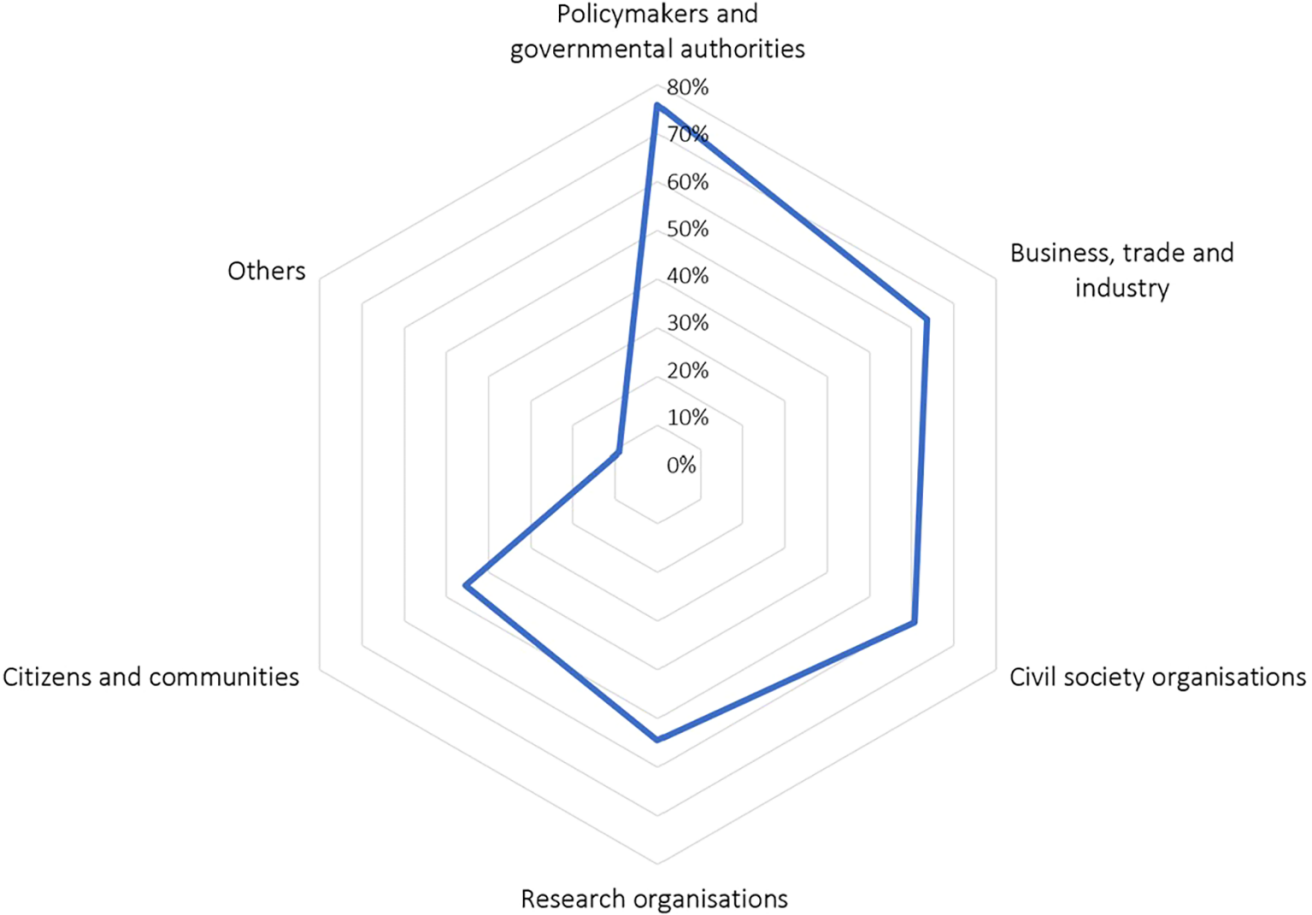
Number of researchers that engaged specific stakeholder groups: Which stakeholder groups are/were engaged in your project?; multiple choices possible, n = 30.

The surveyed researchers involved a range of stakeholders in their projects, as shown in Figure 2. Three-fourth of the researchers involved policymakers and governmental authorities from local to European level, and two thirds engaged business, trade, and industry, as well as civil society organisations. In the survey, researchers also named “other” stakeholders, including small- and medium-size enterprises (SMEs), institutions in the financial and insurance sections, self-employed fisheries, as well as “Friday for Future” activists.

**Figure 3.**
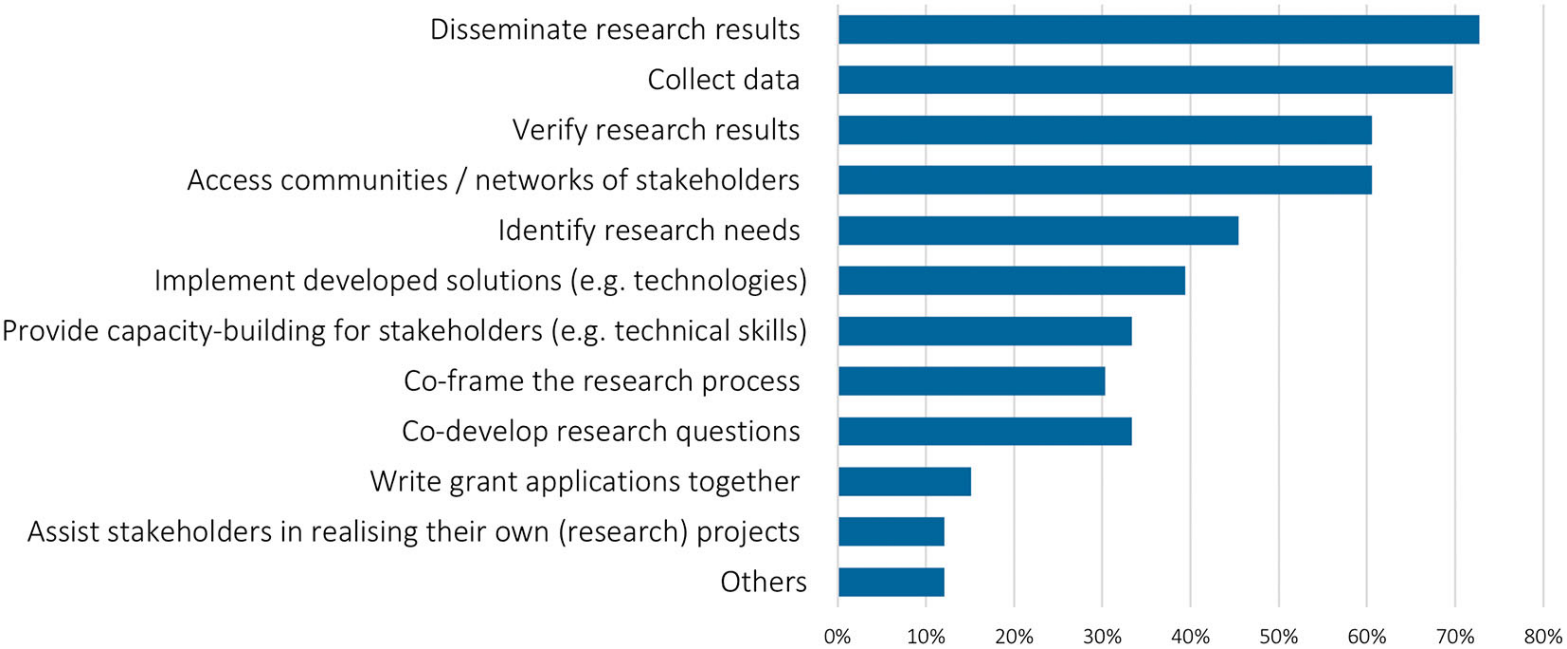
Motives for stakeholder engagement: What is/was the purpose of engaging stakeholders at certain points in the research process?; multiple choices possible, n = 30.

Almost half of the respondents (45%) assessed stakeholder engagement to be crucial for the success of the project; another third saw it as quite important. The importance slightly declined from 2020 to 2022. Researchers had different motives for involving stakeholders in the research, ranging from a pure interest in disseminating research results to even assisting stakeholders in realising their own projects ( Figure 3). One third of the researchers took a co-production of knowledge, or capacity building approach, aiming to, either develop the research process together, what is important in pursuing transdisciplinarity in sustainability science (cf. Mielke et al. 2016) or to directly create a change ‘on the ground’. Researchers also indicated that they involve stakeholders with the specific purpose of generating policy impact, e.g., to co-design policies, sharing learnings for policymaking, or supporting decisions. Compared to 2020, there is no notable difference in answers, except that access/collection of data was more important in the 2022 survey (50% of the projects in 2020, 70% in 2022).

The importance of stakeholder engagement for data collection purposes was elaborated by interviewees that valued the insights and perspectives from stakeholders that are often not reflected in quantitative data. One interviewee said that the *“system cannot be described properly only with statistical data - also in the fishing industry”* (R#5). Furthermore, a recurring theme in the interviews was to involve citizens and communities to increase their “buy-in”, or to find takers for outputs generated in the projects. The two interviewed stakeholders were motivated to participate in the affiliated research projects, because they thematically identified themselves with the topic and they wanted to share their expertise.

### Impacts of COVID-19 on sustainability research

**Figure 4.**
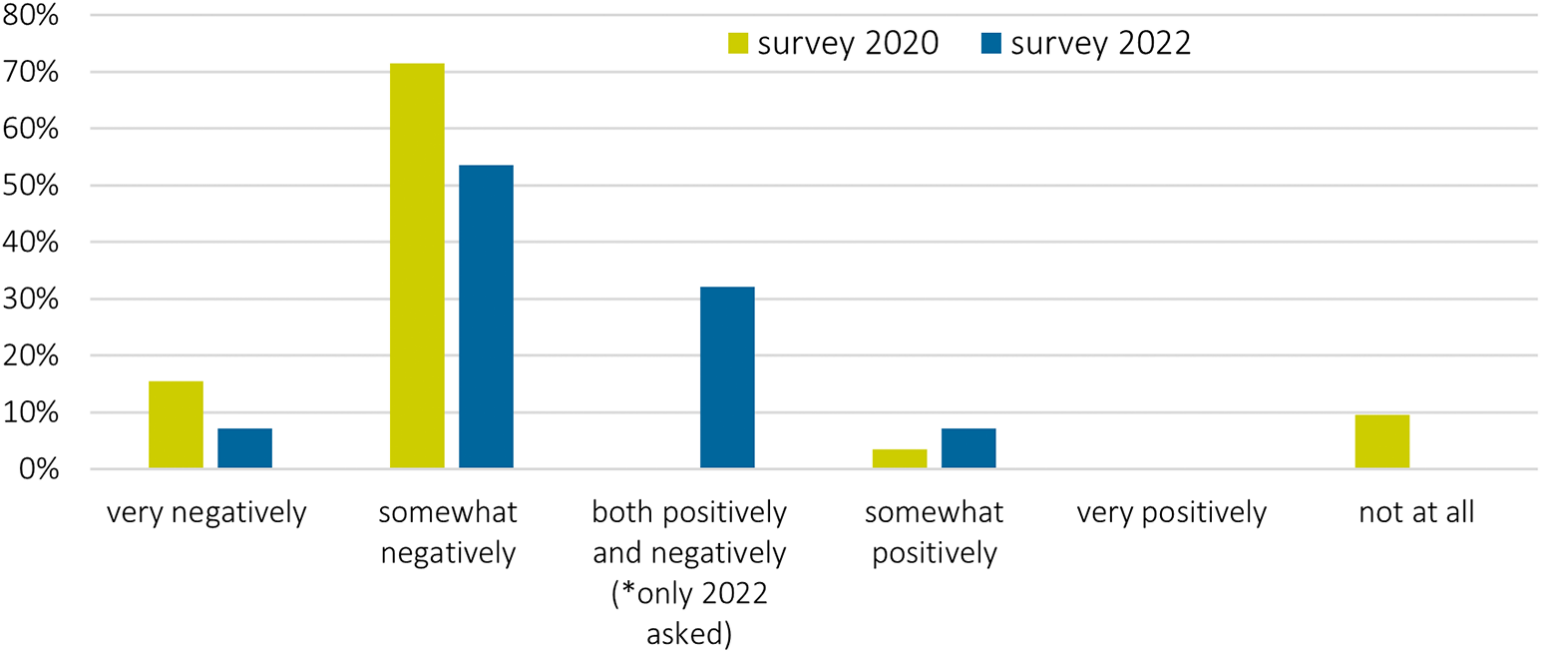
Impact of COVID-19 pandemic on stakeholder engagement: How did the COVID-19 pandemic influence your stakeholder engagement in the project overall?; n = 30 in 2022; n = 84 in 2020.

Overall, the pandemic had a negative impact on stakeholder engagement: more than half of the surveyed researchers saw impacts as mainly negative, in both periods but with considerably more negative responses in 2020 than in 2022 ( Figure 4). Main reasons were that stakeholders could not be reached during the pandemic and that engagement was not as deep as with in-person interactions. For example, one survey participant responded (free text):

*“[W]e could not engage as much as we wanted. It was not possible to meet with some stakeholders (mostly local level) using online tools. That was technically and culturally difficult”* (survey_ID40).

**Figure 5.**
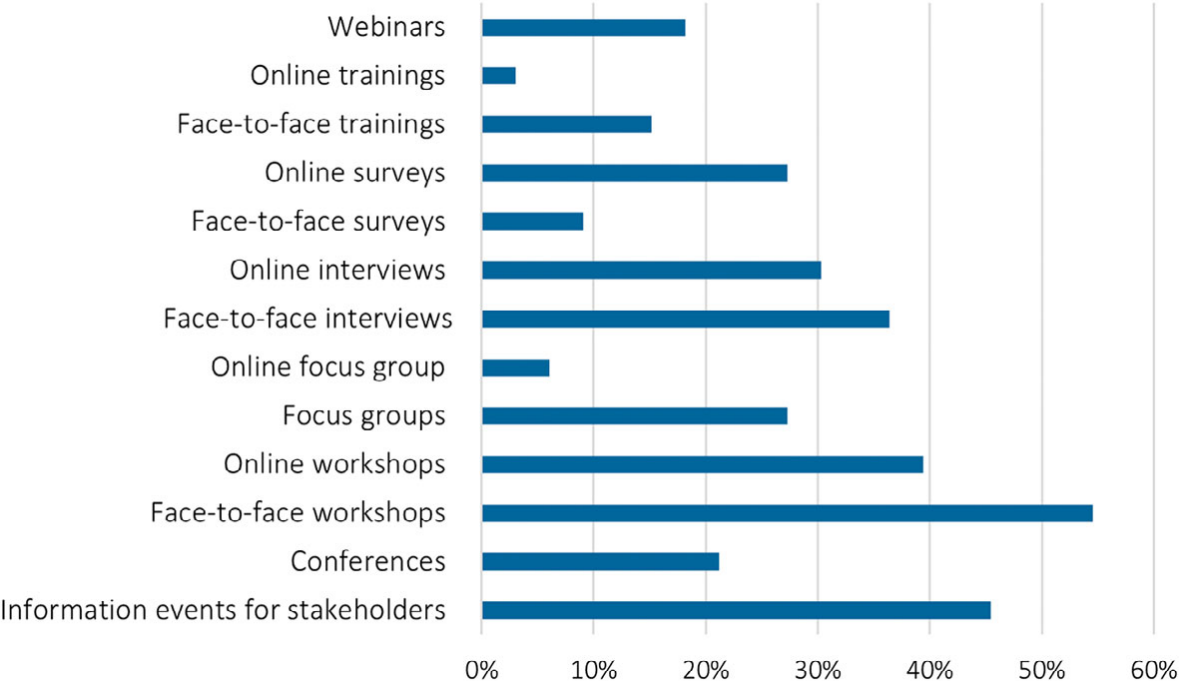
Planned stakeholder engagement activities: According to the original project plan/proposal, which kind of stakeholder engagement activities did you plan to perform in 2020/2021?, n = 30, multiple choices possible.

However, some projects also experienced positive impacts, or were neutrally affected from the pandemic ( Figure 5). These were mainly projects that had already planned for online activities anyway, or that just started to plan their engagement activities during the pandemic:

*“Covid didn’t have that much impact. We did online meetings and training courses anyway”* (R#9).

More than one third of the surveyed researchers had planned for online workshops and almost the same numbers for online interviews and surveys. However, most survey respondents and interviewees had planned to engage with stakeholders physically in 2020/2021 – when COVID-19 lockdowns were most stringent ( Figure 5).

**Figure 6.**
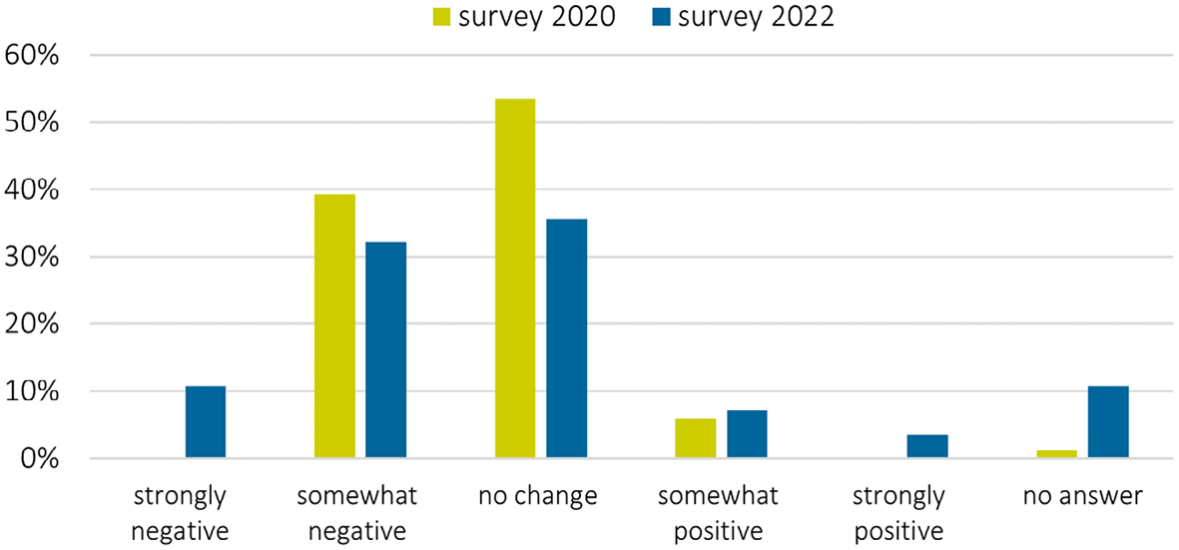
Impact of COVID-19 pandemic on the relationship with stakeholders since the start of the COVID-19 pandemic; n = 30 in 2022; n = 83 in 2020.

One third of the projects experienced both positive and negative impacts ( Figure 6). One survey respondent stated:

*“Stakeholder engagement has been negatively affected by the almost exclusively virtual events, as the important informal exchange among stakeholders is much more difficult, or even impossible. Nevertheless, online events can reach stakeholders who would not be normally reached through physical events”* (survey_ID2).

This quote highlights that positive impacts were related to the scope of the stakeholders that could be reached, whereas it was not possible to deepen the engagement level.

*Process*

The pandemic negatively impacted the engagement of stakeholders in research processes: More than half of the survey respondents agreed that it was harder to familiarise stakeholders with the project goals/objectives and one third agreed that stakeholders had less time, or their priority had shifted away ( Table 1). There was more agreement that exchange with stakeholders had become less frequent, although the received responses about online meetings varied considerably.

**Table 1.** Agreement to the statement about stakeholder engagement; n = 30.

If you consider the stakeholder engagement overall, do you agree with the following statements about stakeholder engagement in 2020-2022 in comparison with the pre-pandemic situation?				
Responses in %	Yes, I agree	No, neutral/ same as before	No, the opposite is true	No answer
It was harder to familiarise stakeholders with the project goals/objectives.	54	46	0	0
Stakeholders had less time.	32	54	14	0
Stakeholders’ priority had shifted away from the project.	32	61	4	4
Exchange with the stakeholders had become more frequent.	18	50	32	0
Stakeholders responded better to remote meetings than physical meetings.	28	32	36	4
Stakeholders got more committed to the project’s activities.	0	57	43	0

Based on our qualitative interview analysis, we found that three dimensions were mainly affected: relationship, coping and preparation.

Relationship

Researchers reported that it was a challenge to establish relationships with stakeholders. Identifying stakeholders and gaining their trust was made more difficult in online engagement:

*“We had taken a lot of time to find people to “introduce” us. Working with fishermen is not that easy; a lot is based on trust […], but then we were prevented from going there to build a relationship of trust“* (R#5).

Some projects wanted to form working or core groups of stakeholders, but many researchers struggled to achieve that.

Our research confirms the first impressions after the COVID-19 pandemic started ( Süsser et al. 2021) that many researchers feel a strongly negative, or somewhat negative, change in their relationship with the stakeholders since the start of the pandemic ( Figure 6). The opinion about the change diverged even more, and less researchers experience no change.

Some researchers reported that it was easier to keep contacts online, also in between in-person meetings, and to exchange materials. After difficulties in the first meetings, stakeholders got used to online events:

“ *It was working very well after a short time […] It enables not to lose engagement, to keep contact, and to develop a lot of things. And everyone is realising that it is very easy to have an online workshop and that you do not even need to travel there”* (R#8).

**Figure 7.**
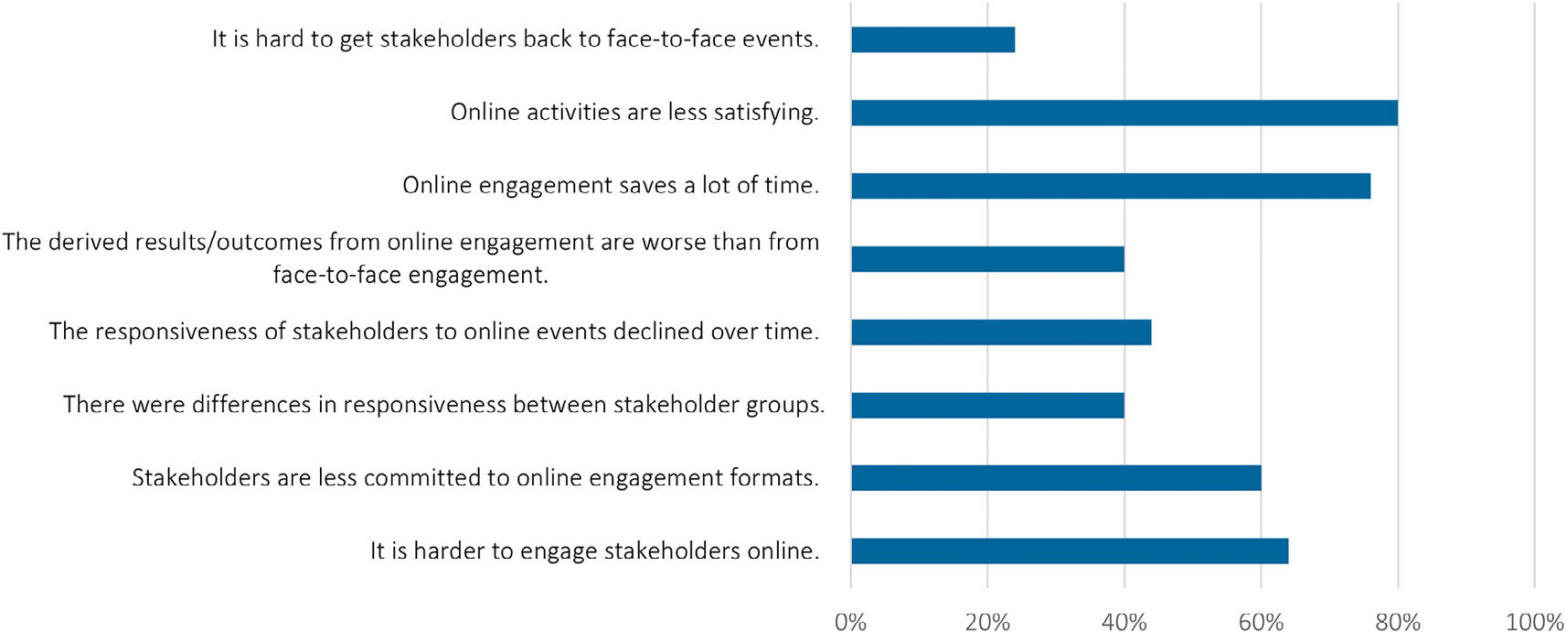
Respondents’ agreement with the following statements about online engagement; n = 30.

For almost two thirds of respondents it was harder to engage stakeholders online, and for them, stakeholders were less committed to online engagement formats ( Figure 7). Often, participation in online events was reported as less active, i.e., many people had their cameras off, were in the listening-only mode, or were doing other things in the background. A survey participant confirmed:

*“Level of engagement was more passive. People with no cameras on, barely speaking. There is a big loss in comparison to the benefits of face-to-face”* (survey_ID40).

A researcher also admitted that *“[it is] too easy to open another window, to open emails. I do it myself”* (R#1). Thus, it was also easier for people to “hide” online, while in in-person meetings th *e “facilitator can rope them in better”* (R#11).

At the same time, anonymity made it easier for people to leave during online events: breaks or shifts to breakout sessions were moments when researchers lost many attendees (R#15). One interviewee confirmed this:

*“If people are in the same room together, like before the pandemic, there is much more buy-in and the willingness to make a good impression when meeting in person. I think it is much easier now to hide behind a closed camera and it is hard to connect when you aren’t able to look someone in the eye. I feel if it was a physical event where people could look each other in the eye and have a handshake beforehand, there would be no issue at all”* (R#1).

We also find that the participation in online events declined over time ( Figure 7). While people *“were more eager to meet online in the beginning, as the pandemic continued, it became more difficult”* (R#9). Interviewees reported that attendance fluctuated, and it was hard to keep contact: *“In general, in Corona there is a greater risk of contact being lost somewhere”* (S#13).

Finally, the relationships to stakeholders were negatively affected because online engagement does not provide the networking activities, informal interactions, and the potential for establishing more personal ties in equal measure. One interviewee underlined that “ *in-person gives connection and sympathy and understanding which is hard to create online. Humanness is critical*” (R#2). However, it was not only harder to build relationships with stakeholders but also within newly formed research consortia. One researcher explained that they received often late or no responses from project partners and that *“[i]f we would have been in the room together once or twice, four times, five times during the project, this just comes way more naturally”* (R#1).

Coping

In 2020, we found that one in six engagement activities was implemented as planned (18%) ( Süsser et al. 2021) – in 2022, it was one in four ( Table 2). After the pandemic started, a dominating strategy was to delay the activities (45% in 2020), hoping that the situation would improve quickly. One interviewee stated that task leaders asked them to wait and delay meetings during the crisis and to meet in-person later in the year; but they did not do any in-person meeting as the pandemic continued (R#12). Similarly, one respondent emphasised the importance of meeting stakeholders at congresses; however, most congresses were cancelled in 2020 and most were even moved to 2022 (R#8). In 2022, substantially less researchers delayed activities in comparison to 2020. As one stakeholder explained: *“because it was not physically possible, and we were not yet technically ready. […] In 2020, a lot of things were cancelled and in 2021, everything was more routine. We always had plan B“* (S#13).

**Table 2.** Overview of engagement activities and impacts of the pandemic on the implementation (numbers), multiple choices possible, n = 30 for 2022; n = 48 for the numbers for 2020 from ( Süsser et al., 2021).

Coping measures: Type of activity:	Planned	Implemented as planned	Socially distanced	Delayed	Cancelled	Format changed	Other
***Information events for stakeholders***	25	0	3	3	4	15	0
***In-person workshops***	29	1	2	5	6	15	0
***Conferences***	9	0	1	3	3	6	0
***Focus groups***	14	0	1	3	4	6	
***In-person interviews***	20	1	2	3	3	11	1 Partially online, partially in person
***In-person surveys***	7	0	1	1	1	4	0
***In-person trainings***	13	0	-	3	3	7	0
***Online workshop***	16	12	0	4	0	0	0
***Online focus group***	1	0	-	1	0	0	0
***Online interviews***	11	9	-	2	0	0	0
***Online survey***	11	9	-	2	0	0	0
***Online trainings***	1	1	-	0	0	0	0
***Webinars***	5	5	-	0	0	0	0
**% of strategy applied in 2022**		23%	6.1%	18.5%	15%	39.5%	
**% of strategy applied in 2020**		18%	9%	45%	18%	52%	7%

Likewise, 2020 and 2022, a minority of some (10) projects implemented the engagement activities with social distancing measures ( Table 2). For example, researchers moved from larger to smaller stakeholder event sizes, or even met stakeholders outside:

*“We even had a face-to-face meeting at someone’s home on the veranda in the fresh air. But that only worked because that was a personal contact from previous projects. There was a great trust and openness there. That was great!”* (R#6).

Also in 2022, many researchers moved activities online ( Table 3). Among the coping engagement formats, online workshops and online interviews were the most common-confirming our findings from the 2020 survey. In-person engagement formats were usually directly replaced by a corresponding online format. For example, planned in person workshops were held online on various video conference platforms, often complemented by interactive tools, such as live polling or whiteboards. Additionally, interviewees reported about online capacity-building events and regional demonstration events:

**Table 3.** Overview of alternative online engagement activities performed, if ‘format changed’ (numbers), multiple choices possible, n = 30.

Type of activity Alternative activities: Originally planned:	Webinar	Online workshop	Online conference	Online focus groups	Online interviews	Online survey	Online training	Other
**Information events**	7	6	2	0	2	1	0	Visit to virtual trade fairs
**Face-to-face workshops**	1	13	0	0	0	0	0	
**Conference**	3	4	4	0	1	0	0	
**Focus groups**	0	2	0	4	0	0	0	
**Face-to-face interviews**	0	2	0	1	8	1	0	
**Face-to-face survey**	0	1	0	1	1	3	0	
**Face-to-face trainings**	1	1	0	0	0	0	3

*“We thought a lot about how to give people the experience of a farm visit that is fun and engaging. For instance, the kick-off meeting was online, so we asked people to send beforehand some pictures of them and a few fun facts about them to break the ice”* (R#14).

Other projects changed the engagement activity ( Table 3). Webinars were less prominently chosen than in the 2020 survey, but were the main choice to replace information events, or congress visits. One interviewee explained that they had to adjust their plan:

*“Involvement was different. Originally, a participatory approach was planned […]. We had to make adjustments early on due to the lock-down. That means face-to-face conversations and also face-to-face interactions were more difficult and instead of workshops planned early on, we then conducted interviews (online)”* (R#6).

Overall, video conferencing tools became much more prominent in engaging stakeholders ( Table 4), but their applications, especially at the beginning, made some stakeholders feel digitally isolated:

**Table 4.** To what extent did you use the following channels for the communication with stakeholders in comparison to post-COVID times?; n = 26.

Channel	Used more often than before	Used same as before	Used less often than before	Did not use it	No answer
E-Mail	10	15	0	1	0
Telephone	3	11	4	8	0
Instant message services (Slack, Whatsapp, …)	3	7	0	16	0
Video conferencing tools	25	0	0	1	0

*“Everyone was digitally somewhat isolated and had to build up their infrastructure on their own - just the question of which tool to work with - with Zoom with Teams […]”* (S#13).

Finally, researchers also had to adapt the event length. Interviewees commonly agreed that long events do not work. One researcher explained that they tried a two-day online event, or long sessions with a break in between – as would have worked in person – but *„doing an event for three or four hours wasn’t possible anymore”* (R#15).

Preparation

Most respondents agreed that online engagement saves time and resources ( Figure 6), also by avoiding travelling:

“ *We had two consortium meetings in person before COVID and our final meeting was kept hybrid. It was an option because it didn’t make sense for people to travel. Moving forward, that is the way we should run projects. Project proposals making winter meetings online and summer meetings in person. We can do it online, cheaper, climate-relevant. It is good to be more critical and ask what is the value of in-person versus online. Taking four days off of their daily work for a project meeting is a hurdle”* (R#2).

Simultaneously, preparing for online engagement may require more effort:

*“You should over prepare when going online. Over-prepare on content, also be very aware of the timeframe. Be flexible. Take into account people’s capabilities. Be respectful of other people’s time”* (R#7).

One stakeholder highlighted the capacities needed to implement online event successfully:

*“There are really good things that you can use to structure digital communication very well. […] I think you need certain resources, you need the (wo)men-power, the experience, to have dealt with it very intensively in advance. That is also a challenge. The preparatory work you have to put into it is already very big and you can’t underestimate that”* (S#13).

In addition, conducting individual interviews was reported to be more time-consuming than the implementation of one workshop, also as *“you need to repeat several times to generate the same impacts as if you would meet the people in person”* (R#8). Further, it takes more time to explain complex topics online. One interviewee shared the experience that it was difficult to present complex approaches behind mathematical models online; it did not work out because they were not able to explain it, stakeholders could not engage in breakout groups (R#15).

*Outcomes*

The pandemic also had impacts on the outcomes of the research as well as on the outcomes of the stakeholder engagement specifically.

Outcomes of research

**Figure 8.**
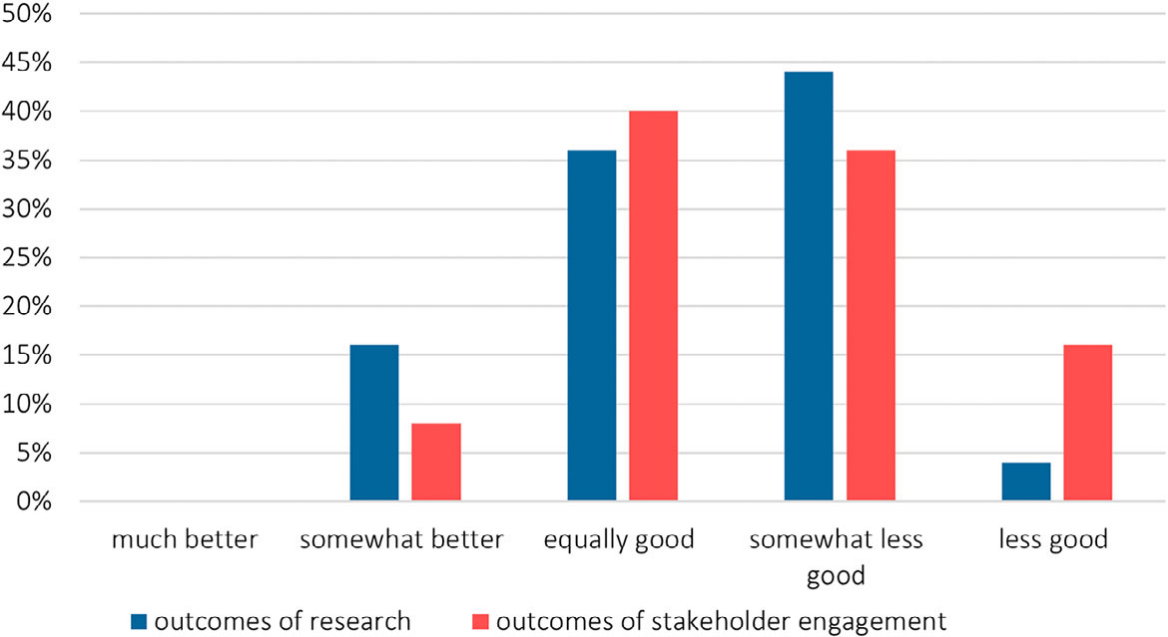
Impact of the pandemic on the outcomes of the project and stakeholder engagement, n = 25.

**Figure 9.**
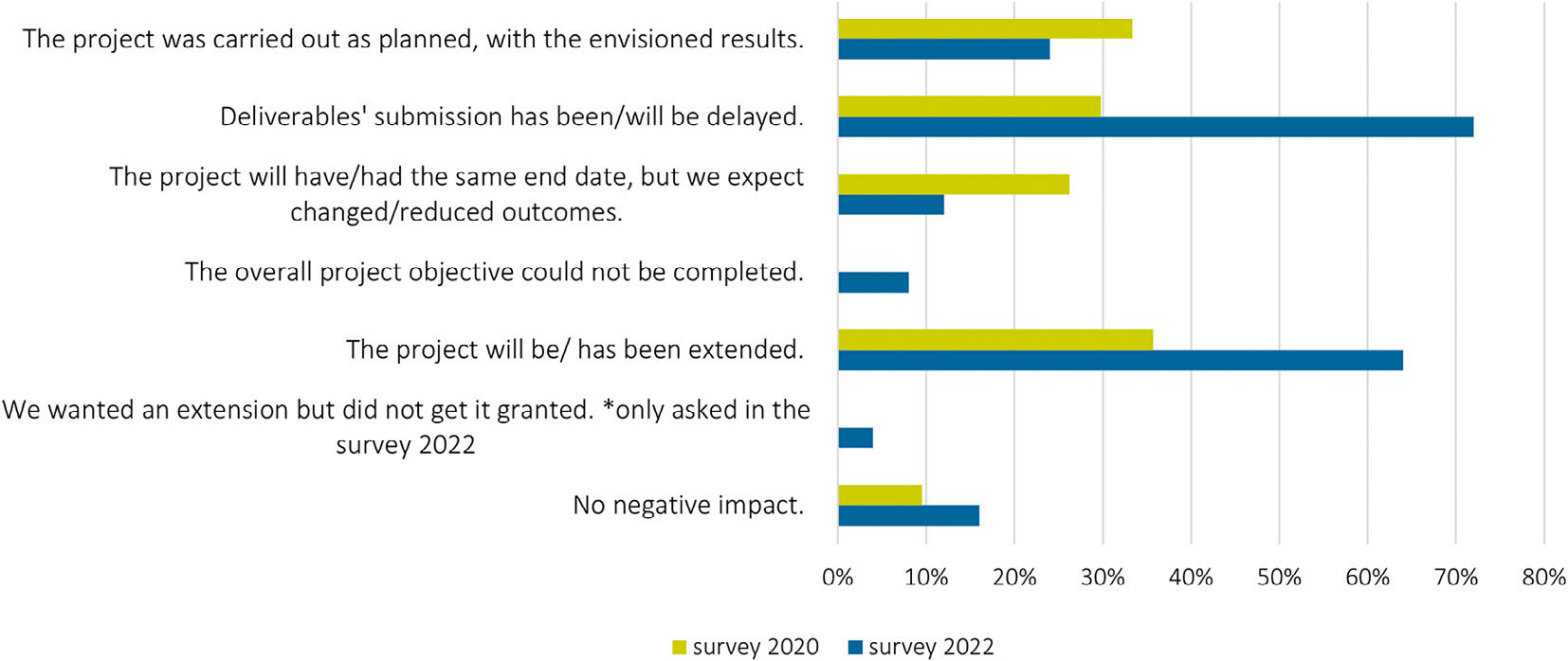
Impact of changes in stakeholder engagement on the overall proceedings and results of projects; multiple choices possible; n = 30 in 2022; n = 83 in 2020.

Around half of the researchers reported that the outcomes are (somewhat) less good ( Figure 8), while less than one third of the respondents stated that the project was carried out with the envisioned results ( Figure 9). The reasons given were mainly related to the depth of the generated results:

*“We did mainly get superficial results, no deep ones, such as a common vision, policy recommendations, implementation plans, …”* (survey_ID63).

One interviewee reported that they were able to “tick the boxes” of the Grant Agreement, but not in effective ways.

*“It’s doable, but it’s a difference in quality. It’s like a Michelin star meal compared to plain rice. Both will feed you, but you’d rather have the higher quality option. You can do everything online, but it isn’t as nice. I came into the project motivated to create the Michelin star meal, but it ended up bare minimum of rice. It’s de-motivating, you’re less willing to engage with project partners and stakeholders. By properly writing clear quality indicators, it might be a way to keep the motivation higher*” (R#1).

The impacts of the pandemic caused projects’ extensions that became even more evident in the 2022 survey ( Figure 9), with two thirds of the projects extended.

No negative impacts and even better outcomes were reported by minority of respondents, one of them mentioning about different opportunities:

*“Focusing on outcomes or outputs, I have to admit that the shutdown in activity due to COVID was very useful to get some writing done. While we lost out on some engagement, we were lucky that the projects were nearing their end rather than starting out. […] It gave time for reflection, something we had struggled to find before. Of course, if we hadn’t already been working together for two years, and I didn’t have two rounds of community meetings done, the impact would have been very bad”* (survey_ID29).

Outcomes of stakeholder engagement

Most respondents agreed that online activities are less satisfying ( Figure 7). Online engagement often misses ‘lively’ interactions, as one interviewee explained:

*“But the spontaneous, the personal, the blurting out, the creative development, the bending over a paper in a room and painting together, that’s not possible. You already have that distance digitally”* (R#6).

As a result, some researchers emphasised the lower quality of data derived from online engagement:

*“There are a lot of tools for getting people to fill in workshop exercises online, but I find that the quality of data coming out of online engagements isn’t as good as in-person. Some people aren’t comfortable navigating the tools and it just doesn’t work as well as sitting around a table together to put down post-its or fill-in a map”* (survey_ID29).

In contrast, other researchers reported better outcomes from the stakeholder engagement, which were mainly related to an increased participation and the collection of more diverse stakeholder insights. Thus, researchers could gain important insights, albeit “ *less representative”* results (survey_ID35).

Furthermore, deliverable submissions were delayed – in 2022, 70% of the survey participants reported pandemic-related delays in comparison to 30% in 2020 ( Figure 8). For example, due to delays in results from stakeholder input, the results could not or only partially be considered in the project context:

*“Due to the COVID-related problem of talking to them (fishermen), our part was postponed and could therefore be included in the modelling too late or not deeply enough. […] Modellers could get on with their work nicely, but we couldn’t deliver a different perspective (from stakeholders) for a long time”* (R#5).

### When to stay offline, when to go online?

In the interviews, we identified three key influence factors deciding whether to stay offline or go online: (i) intention/ purpose of the stakeholder engagement, (ii) the engaged stakeholder group, and (iii) previous relationships. As one interviewee put it: “ *a different target group, different topics, and different intention of the project/co-creation would need a different format, but it is hard to know”* (R#2).

*Online vs. offline depends on the objective of the stakeholder engagement*

First, the online vs. offline choice depends on the objective of engagement: “ *What is the aim? What do you want to get out of stakeholder engagement?”* (R#9). In case researchers need to gain expertise fast, purely want to report results, or get feedback, online engagement was often sufficient. In contrast, deeper exchange needs in-person interaction:

*“If you need quick expertise, then a short phone call or a Zoom interview is incredibly effective. When it comes to exchanging deeper perspectives, personal things, when it comes to building trust, I think it makes a lot of sense in project contexts to meet in person, especially at the beginning”* (R#6).

Offline experiences are of specific relevance if you want to get impressions “from the ground”:

*“The problem with online meetings is that you cannot show the demo site and socialise. People would like to have a glass of wine but that can’t happen online”* (R#7).

In addition, one researcher shared that you may miss “off-the-record information”, shared in a more informal setting, which would otherwise help in interpreting the interviews:

*“And when we did the interview in person, we also found that when the recorder was switched off, another huge bundle of information came out”* (R#5).

Thus, offline engagement seems to be essential if a deeper understanding of people’s contexts is crucial.

*Online vs. offline depends on the engaged stakeholder group(s)*

Second, the online vs. offline choice depends on the stakeholder group to be involved. Around 40% of the survey’s respondents perceive differences in the responsiveness between stakeholder groups ( Figure 6). Researchers noted that some stakeholders could be better reached online, especially for engagement with policymakers or with industry actors. Interviewees explained that it is *“sometimes […] easier to reach people that are very busy, because they can just take a short time slot in their agenda”* (R#8), or *“[i]ndustry appreciated online because they didn’t have to travel”* (R#9). One of the stakeholders illustrated a difference between different actors on a different example:

“ *If stakeholders are used to having a lot of meetings, such as banks, then you can easily integrate online meetings. However, real-estate investors prefer physical meetings*” (S#13).

In contrast, other researchers reported less engagement and interest. There was a common agreement that *“citizens are hard to engage only online”* (survey_ID37). Nevertheless, one survey respondent shared an insight that incentives and the mediator can motivate citizens to participate in online survey:

*“People are receptive to online surveys with prize money awards (100€ x 5 participants), when the local authority is the communicator”* (survey_ID61).

One fourth of the survey respondents stated that they could not reach some stakeholders online, which can lead to a selection bias. These stakeholders included mainly actors at a local level, such as *“local communities with limited internet access and technological skills”* (survey_ID40), or *“residents who do not have internet or barely use internet”* (survey_ID61). One survey participant mentioned that online engagement can exclude certain people:

*“The community group I worked with didn’t like the idea of hosting online workshops as they felt it would exclude a lot of people who had attended the in-person meetings that were older and may not have access to laptop/internet”* (survey_ID29).

Differences in possibility of reaching out to different stakeholders have also a geographical or cultural dimension, as one interviewee said:

*“There are differences in countries: in UK, or in Netherlands, farmers are used to using laptops and the internet. In the Southern Europe, farmers aren’t that used to the internet”* (R#11).

We found that technical abilities were a barrier, especially in the beginning of the pandemic, as everyone had to learn the new tools. Thus, interviewees also raised the concern that the online community is a subset consisting of people who like using online tools (R#11).

For certain stakeholders it seems to be crucial to stay offline, as they expect to meet researchers in their “environment”, what can bring benefits to the researchers, too:

*“If it’s really about someone like the fisherman, then I would say that’s a target group where it’s more productive to meet on site. For two reasons: You get a better feel for the environment when you meet with someone in their hall, or on their boat. The second reason is that it’s easier to establish a personal connection”* (R#5).

*Online vs. offline depends on the previous relationship to stakeholders*

Third, the online vs. offline choice depends on the strength of ties already established with the stakeholders and project partners. One interviewee underlined:

*“It is also a big difference if you already have a stakeholder group, they all know each other and have met in real life […], instead of just having them mixed together for a moment at an online event”* (R#15).

Our findings indicate that an in-person kicking off event where participants get to know each other and then having online interactions boosts the interpersonal collaboration and trust. The same applied to researcher-stakeholder interactions and projects’ consortia:

*“Online was best where the projects had started before, i.e., where you already knew the people, you already had a project meeting”* (R#5).

We also found that online engagement is often much more disciplined and formal. One interviewee stated that “protected spaces” are missing:

*“You don’t have protected spaces anymore. They all sit in a meeting and therefore everyone is very reduced with expressions”* (R#6).

Thus, we find the need to stay offline, if an open exchange is wanted, potentially also in smaller groups.

### Future perspectives for stakeholder engagement

**Figure 10.**
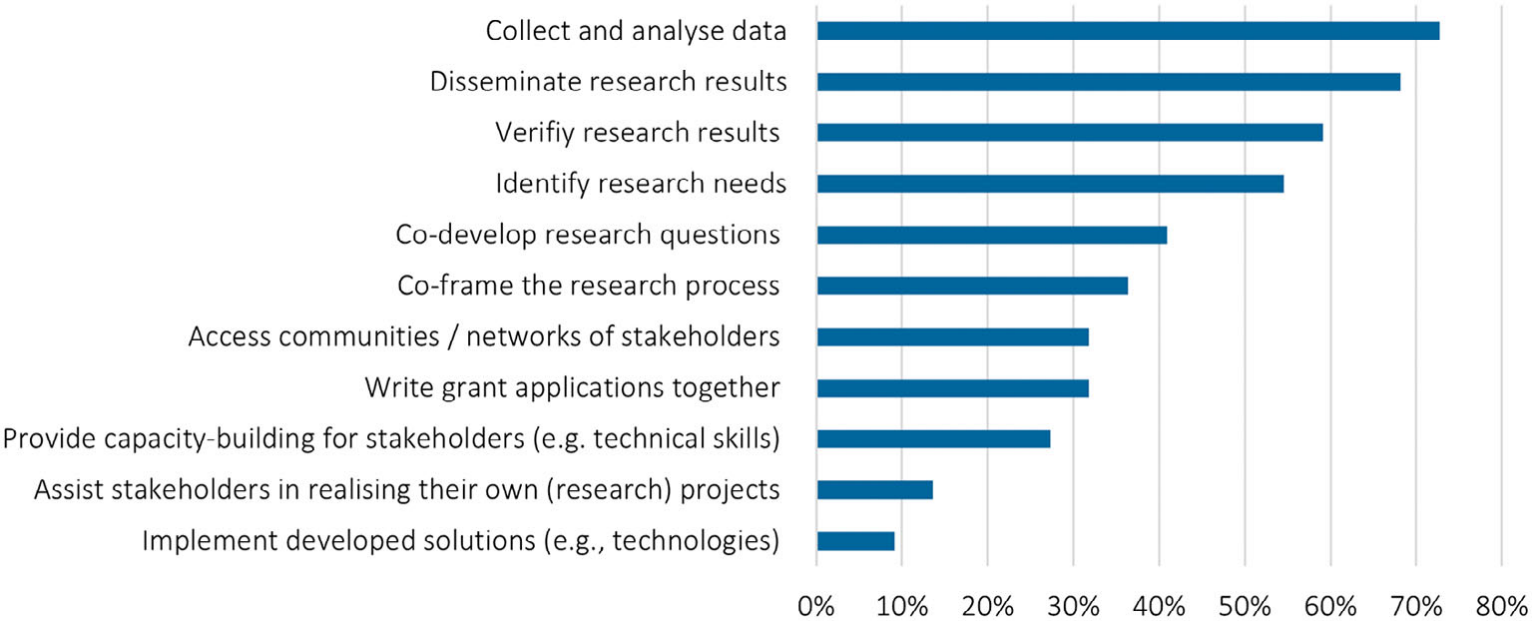
Motives to engage stakeholders online: For what purpose will you continue involving stakeholders online?; multiple choices possible; n = 24.

The experiences with online engagement formats are mixed, but by far most researchers will continue online stakeholder engagement: 92% survey respondents state they will continue doing online stakeholder engagement. In most cases, the main purpose of such future online engagement is to collect data, disseminate research results, verify results and identify research needs ( Figure 10). More intensive stakeholder engagement, in comparison, is much less prominent to be performed online in the future, again indicating that deeper stakeholder participation requires in-person engagement.

**Figure 11.**
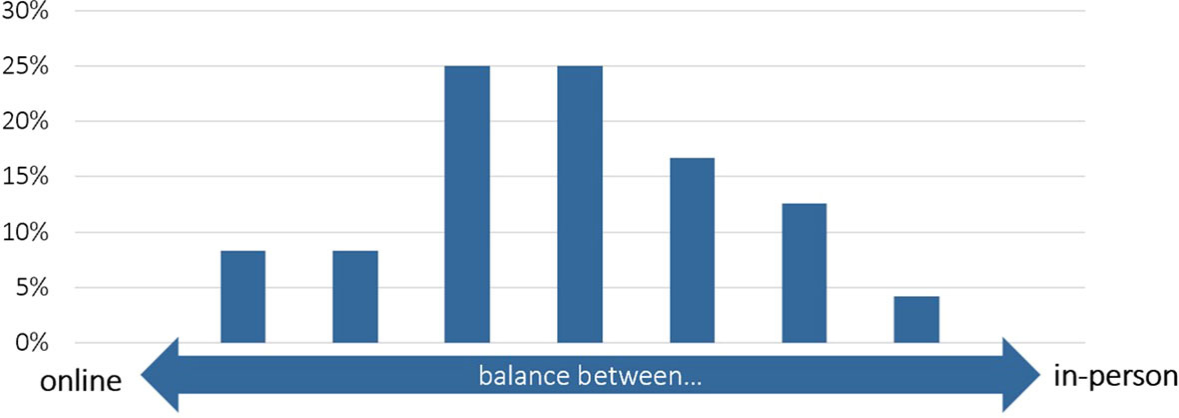
What balance between online and in-person engagement do you plan to strike in the future?; n = 24.

No survey respondent plans to strike for fully online, or fully in-person events in the future ( Figure 11). Instead, the majority plans a mix of online and in-person formats:

*“People now you can do both, to meet virtually and in presence […] before, virtually meetings were not that used”* (R#8).

*How researchers want to engage stakeholders*

We find that most researchers want to continue with online engagement alongside physical engagement. As one interviewee put it: *“Only online is no good, lots of travel is also not good“* (R#9). Researchers value personal contacts and interactions.

For the internal project management, researchers want a reduced number of in-person meetings – two in person, otherwise online. One researcher reported that, while planning the final project event in person, stakeholders, and even consortium members wanted to remain online and were not interested in travelling for the final project event (R#1).

Researchers made different experiences with online engagement formats, with interviews, surveys and workshops scoring highest as the most suitable online formats and the ones most likely to remain in the future ( Table 5).

**Table 5.** You stated that you chose alternative engagement formats due to the COVID-19 restrictions. If you consider the goals of the stakeholder activity you wanted to perform originally, how suitable were the following formats as a replacement (if performed)?; n = 24.

Channel	Very unsuitable	Rather unsuitable	Rather suitable	Very suitable	Not performed (yet)
Webinar	3	3	5	3	6
Online interactive workshop	1	3	11	3	2
Online conference	4	3	4	1	8
Online focus group	0	6	5	0	9
Online interview	1	1	3	10	5
Online survey	0	2	4	9	5
Online training	1	1	5	0	13
Hybrid events (with face-to-face and online participation at the same time)	5	4	8	1	2

Hybrid events were for many a completely new format and researchers made mixed experiences. A survey respondent explained:

*“Hybrid formats are a “maybe” for me since they are even more time-consuming, and they create groups (the ones in the room and the ones attending online), but of course they have the advantage of not excluding anyone (completely)”* (survey_ID34).

Interviewees recognised the time effort behind hybrid events, but also confirmed the advantages for inclusivity:

*“Hybrid formats are the new frontier of how to make it work well and inclusive, that everyone can hear each other and see each other well”* (R#2).

*How stakeholders want to be engaged*

Stakeholders want to participate in online formats as complementation but not as full replacement. Stakeholders prefer a balance of online and physical meetings and believe this format will continue in the future:

*“[…] because you always have the alternative. You say: “Listen, we should really meet once in a while, but these and the certain milestones, we can also meet digitally. We don’t have to plan another hour’s travel time for that”* (S#13).

If a physical engagement is to be organised, stakeholders prefer shorter and more time-efficient events. One researcher shared their experience on their first in-person event after pandemic:

*“Our first in-person event was in Denmark two weeks ago and we scheduled it as a two-day event, and we had every minor registration. And then we changed it to a one-day event and then we got more than double the registrations we already had”* (R#15).

Stakeholders do not see the need to always meet in-person. Interviewees shared that some associations and authorities asked if meetings could be done online (R#5), or that it was hard to get policymakers attend physical events even if the event was designed to fit transport connections and minimising travel time (R#15).

When it comes to specific online tools, stakeholders seem to generally prefer Zoom (R#7). A plethora of online tools were scrutinised during the pandemic, such as virtual whiteboards and online polling, and some stakeholders very much enjoyed new online tools:

*“I have worked with some formats that have also added real value”*, explained one stakeholder (S#13) *, “because it ensures that nothing gets lost”.*

Interviewees generally assessed miro and mural boards to be difficult tools for stakeholder interactions and instead, a google document might be a better way to capture feedback from people.

When it comes to the frequency of interactions, stakeholders generally want to stay informed about the timelines and *“get a feeling of what further steps are planned”* (S#13). Furthermore, they want to be informed about the final results and, specifically about how their inputs added value to the project. One researcher said:

*“I think […] the need of choosing alternative tools, helped everyone to understand that you need to be very concise and explain to the stakeholders what we need and how they can contribute; show that they can contribute to the research and that their feedback is also important […]”* (R#8) *.*

Physical events with hybrid online options are generally fine for stakeholders but mean an additional technological and coordination challenge. Another stakeholder raised issues like:

“ *Who will actually show during physical meeting plans? How will we improve hybrid meetings for the future? How do we create and improve interactivity? Hybrid is hard to change when clients see the benefits and adapt. Two years ago, 90% of our client meetings were physical, I believe that the move from completely physical to hybrid online will stay this way*” (S#3).

## Discussion and conclusions

Our research on the long-term impact of the COVID-19 on stakeholder engagement in European sustainability research showed that the pandemic negatively impacted project activities in the short term but has predominantly *improved* stakeholder engagement in the long run. This is mainly due to five reasons: (i) the pandemic has encouraged people to act differently; (ii) online activities have expanded the engagement portfolio; (iii) online engagement activities turned out to be time and resource savers; and (iv) the pandemic has raised awareness about the nuances of meeting stakeholders in person (and when not to do it). We discuss these points and their implications below.

First, the pandemic forced researchers to quickly find alternative formats and tools for engagement, which made them think beyond standard practices and look for innovative solutions. This spurred not only immediate innovation in science-stakeholder interactions, but also triggered long-term, lasting changes, as existing practices were questioned, including the value of and need for in-person meetings, and how to engage stakeholders more effectively. The coping measures taken showed that both researchers and stakeholders are adaptable and flexible and largely open to change and reap benefits of online tools. When researchers were forced to learn to use online formats and tools, new interactive tools, such as breakout groups or live voting, proved useful. Thus, the pandemic showed that online engagement is a real option.

Second, online engagement expanded the engagement portfolio. The pandemic brought a surge of innovation to digital communication practices. Today, three years after the pandemic started, digital formats have become standard tools expanding the possibilities for engagement and allowing researchers to choose appropriate formats depending on the context. Different stakeholder groups require different forms of engagement, and therefore online and offline formats are differently appropriate depending on the specific stakeholders and their capacities, the depth of previous collaboration and the goal of the engagement process.

While it is impossible to fully replicate the character of in-person meetings in online engagement formats, there are possibilities to facilitate a friendly, more “personal” relationships between the participants, such as different ice-breaking activities. A different prerequisite to foster a meaningful online stakeholder engagement is to make sure about constant and high-quality communication before and after the engagement activity that would allow to share the details about the activity and provide space to share feedback. Despite substantial context-dependency of stakeholder involvement in sustainability research, the meaningful engagement requires a careful consideration of the involvement’s objectives and corresponding qualitative aspects, such as the stage of the research (or project) or the desired level of informal interactions allowing to realise given objectives. Our results suggest that the expanded engagement portfolio gives researchers different alternatives which format would be the most suitable to achieve the research goals.

Third, our results show that online events are time and resource savers, despite the potentially longer preparation time for organisers of online activities. Stakeholders and researchers consider more carefully whether a more resource-, time- and emission-intensive face-to-face event is required – as also suggested by Klöwer et al. (2020). Online engagement activities can also provide new opportunities for the involvement of stakeholders from different countries or even continents, as it requires no financial resources while bridging geographical distances. In practice it may remain hard to include everyone across time zones. We expect a more rigorous reflection on the need for in-person interactions and the necessary length of them, and whether, in some cases, online activities can offer a more inclusive alternative.

Fourth, the pandemic has raised awareness about how important and necessary it is to still meet in person. Although online engagement worked better than most researchers and stakeholders initially thought, our findings suggest that in-person activities still guarantee a deeper connection and exchange between people in comparison to online formats. This is particularly important in co-creative research, for which stakeholders must be very closely involved in the research process and empowered to take actions. In addition, much emphasis was put on casual conversations during or after official events, and on informal insights that are lost in online-only activities. Thus, researchers and stakeholder want, likewise, a mix of online and offline engagement in the long run.

Our research has provided qualitative insights into the impact of the pandemic on stakeholder engagement in various sustainability research projects and the implications for the long-term future. Thus, these findings can provide recognition and important guidance to researchers and funding agencies on the opportunities, but also challenges for meaningful stakeholder engagement in research. However, the data gathered may not be representative of the entire research and, especially, stakeholder universe of perspectives. In particular, we were only able to interview two stakeholders and therefore stakeholder insights remain underrepresented. This is mainly due to the data security of the research projects, as they could not provide us with information about the stakeholders involved. In addition, we struggled to recruit researchers to participate in our survey and even received no responses at all from stakeholders. This may, ironically, be related to an arising ‘online fatigue’ in 2022 and the new opportunities to meet in person again. Future research could build on our work and perform a more project-specific and continuous evaluation of the effectiveness and success of online engagement and its limitations. In addition, future research could analyse the impacts of the pandemic on research funding.

### Ethical considerations

The research was conducted in accordance with the ethical requirements and guidelines of the SENTINEL project (Deliverables 11.1 and 11.2), which follows the guidelines of the European Commission. We used an ethically sound methodology for data collection and processing in this study, under the guidance of the Institute for Advanced Sustainability Studies (IASS), now the Research Institute for Sustainability – Helmholtz Centre Potsdam (RIFS), Data Protection Service. This was supported by bilateral data protection agreements.

### Consent to participate

Interviewees agreed to our data protection standards through a signed consent form, and survey respondents agreed to our data protection standards through a GDPR disclaimer and by participating in the survey.

### Consent to publish

The results from the survey and the interviews have been de-identified in accordance with the Safe Harbor methods, ensuring that individuals cannot be identified.

## Data Availability

The questionnaire and the interview guidelines can be accessed on Zenodo ( 10.5281/zenodo.10812990) under the Creative Commons Attribution 4.0 International. The interview results and survey data underlying this study are not openly available to protect the anonymity of participating individuals and projects. Given that we asked for many details of the projects, like funding source, duration etc., we cannot guarantee anonymity. For further COVID-19 research and the replicability of the research in another study context, access to semi-anonymised data can be granted. For further information on the data please contact Diana Süsser (diana(a)ieecp.org).

## Footnotes

^1^
We use “COVID-19”, “Corona” and “pandemic” interchangeably to refer to the outbreak of the coronavirus disease in early 2020.
