# Supplementary material for: How the COVID-19 pandemic changed stakeholder engagement processes in sustainability research in the long-term
Source: F1000Res. 2024 May 7;13:458. [Version 1] doi: 10.12688/f1000research.145238.1 (PMC11320055; doi:10.12688/f1000research.145238.1)
Supplement: Peer review history

# Reviewer report: Reviewer response for version 1

**DOI:** 10.5256/f1000research.159156.r303915

**Published:** 2024-09-03

**Authors:** Ines Omann

**Affiliations:** 1 Vienna University of Economics and Business, Vienna, Austria

Thank you for this well structured and clear paper, giving a good overview of pros and cons of online participation vs. physical meetings.

 Here are some comments and suggestions.

 - I wonder, whether your analysis is valid only for sustainability research projects or if they are valid for any transdisciplinary applied research projects. If not, please explain how the results are linked to sustainability.-

 - The results are well summarized. It is not fully clear how you came to your conclusions. If I may play the advocatus diaboli: the results are quite predictable and straightforward. Are there any surprises that found?

 - There are some more detailed comments in the pdf.

 - Please add in the appendix the interview guideline and the survey.

Is the work clearly and accurately presented and does it cite the current literature?

Yes

If applicable, is the statistical analysis and its interpretation appropriate?

Yes

Are all the source data underlying the results available to ensure full reproducibility?

Partly

Is the study design appropriate and is the work technically sound?

Yes

Are the conclusions drawn adequately supported by the results?

Yes

Are sufficient details of methods and analysis provided to allow replication by others?

Partly

Reviewer Expertise:

socio-ecological transformation, climate change, stakeholder engagement, public participation

I confirm that I have read this submission and believe that I have an appropriate level of expertise to confirm that it is of an acceptable scientific standard, however I have significant reservations, as outlined above.

## References

[1] : Stakeholder engagement and decarbonization pathways: Meeting the challenges of the COVID-19 pandemic. *Frontiers in Sustainability* .2023;3: 10.3389/frsus.2022.1063719 10.3389/frsus.2022.1063719

# Reviewer report: Reviewer response for version 1

**DOI:** 10.5256/f1000research.159156.r303919

**Published:** 2024-08-12

**Authors:** Heta Leinonen

**Affiliations:** 1 Tampere University, Tampere, Finland

Dear Authors,

 Thank you for the opportunity to review this highly interesting and topical article!

 In the article, the authors propose novel insights into how the COVID-19 changed stakeholder engagement (SE) in sustainability research.

 In the following, I give my main comments on the article point by point.

 1. Theoretical considerations

 1.1 Please define (more reliably) the concepts that you use. For instance,

- stakeholder (e.g., Freeman, R. E. (1984). *Strategic management: A stakeholder approach*. Pitman.)
- SE (e.g., Greenwood, M. (2007). Stakeholder engagement: Beyond the myth of corporate responsibility. *Journal of Business Ethics*, 74(4), 315–327. https://doi.org/10.1007/s10551-007-9509-y) In the article, does SE mean the same as SE process and/or stakeholder involvement? Further, in this article, does SE include the aims, activities, and impacts of SE or do you focus only on the activities? In addition, it would be interesting to know how the interviewees and respondents understood SE: were they given background information on what is meant by the concept?
- stakeholder-based research
- sustainability (research)

1.2 You write about the same phenomenon with many different concepts, e.g.,

- SE format, activity, tool, portfolio, practice, form, interaction… (In addition, in the article, do they mean the same as “project activities”?)
- COVID-19, pandemic, COVID-19 pandemic, Corona, coronavirus disease…

When you have chosen the concept, please use it throughout the article. When consistently using only one concept, you would not need, e.g., the footnote on p. 3: “We use “COVID-19”, “Corona” and “pandemic” interchangeably to refer to the outbreak of the coronavirus disease in early 2020."

 1.3 Based on previous points, please reconsider the title “How the COVID-19 Pandemic Changed Stakeholder Engagement Processes in Sustainability Research in the Long-term”. Could it, e.g., be “How the COVID-19 Changed Stakeholder Engagement Activities in Sustainability Research”?

 1.4 In the Discussion and Conclusions, theoretical contributions are missing.

 2. Methods

 2.1 Great that you state the research question at the beginning of the article (p. 3).

 2.2 As you had only two stakeholders in the interviews, and all the other interviews and the survey were for researchers what if you include only the researchers in this study? For the reader, it would clarify the scope of the research.

 2.3 Could you include a table on interview data (incl. e.g., date, length (min), and transcripted pages)?

 2.4 What language were the interviews in, i.e. are the quotations translations? Please check, if, e.g., these quotations are correct: “People now you can do both, to meet virtually and in presence" (p. 15) and "Our first in-person event was in Denmark two weeks ago and we scheduled it as a two-day event, and we had every minor registration" (p. 16).

 3. Results

 3.1 While reading this part of the article and interpreting the visualizations related to it, at some point, I was unsure where the survey was discussed, from which year (2020 or 2022), and whether only the interviews or the synthesis of the survey and the interviews were discussed. In other words, the guidance and reader-friendliness of this section could be improved.

 3.2 On p. 15 under the heading "How Researchers Want to Engage Stakeholders" you mention “internal project management”. That made me think about the limitations of the study and the previously mentioned need for the definition of stakeholder. Please, define stakeholder at the beginning of the article and also define to what extent the SE _within_ the research groups is within the scope of this study.

 3.3 At some places, you have strong statements that could be softened (e.g., p. 6: "Overall, the pandemic had a negative impact on stakeholder engagement" and p. 7: "The pandemic negatively impacted the engagement of stakeholders in research processes") based on your results.

 3.4 On p. 7, you mention "three dimensions" under the heading "Process". What do you mean by dimensions and process? What are the other dimensions that were not affected?

 3.5 On Table 2 (p. 10), replace - with 0.

 4. Spelling and Grammar

 4.1 Please use – in between numbers instead of -.

 4.2 In terms of repetition, please check the following sentence on p. 14: "One of the stakeholders illustrated a difference between different actors on a different example”.

 I offer you my best wishes with the editing and future research!

Is the work clearly and accurately presented and does it cite the current literature?

Partly

If applicable, is the statistical analysis and its interpretation appropriate?

I cannot comment. A qualified statistician is required.

Are all the source data underlying the results available to ensure full reproducibility?

Partly

Is the study design appropriate and is the work technically sound?

Yes

Are the conclusions drawn adequately supported by the results?

Yes

Are sufficient details of methods and analysis provided to allow replication by others?

Partly

Reviewer Expertise:

Stakeholder engagement, a circular economy, sufficiency

I confirm that I have read this submission and believe that I have an appropriate level of expertise to confirm that it is of an acceptable scientific standard, however I have significant reservations, as outlined above.

## References

[1] : Stakeholder Engagement: Beyond the Myth of Corporate Responsibility. *Journal of Business Ethics* .2007;74(4) : 10.1007/s10551-007-9509-y 315-327 10.1007/s10551-007-9509-y

# Reviewer report: Reviewer response for version 1

**DOI:** 10.5256/f1000research.159156.r303921

**Published:** 2024-08-07

**Authors:** Neetha Shagan Morar

**Affiliations:** 1 HIDRU, South African Medical Research Council, Durban, South Africa; 2 South African Medical Research Council, Durban, South Africa

Thanks to the authors for this valuable, relevant and important paper put together on stakeholder engagement (SE)in sustainability research. The descriptive analysis is well explained with several tables and highlights the influence of the COVID_19 pandemic on SE. The  following comments are to enhance the publication on the  importance of SE during  and post covid-19. It also includes a few points of clarification and use of terminology.

 Title: Please indicate from a researchers perspective and where was the project in the title as there were only two stakeholders interviewed from the North areas. It is recommended that a follow up study may include more stakeholders and not only researchers responses as they play a critical role in SE.

 Abstract may be more valuable if it included the sample size and state that pandemic refers to COVID-19 or add term when referring  to pandemic.Fo consistency refer to sustainability research and SE vs stakeholder based research. 

 Introduction is well explained and includes a methods section that could be moved to the relevant methods section.  Please consider adding the new complexity issues that COVID-19 added with a reference in paragraph one, Stakeholder based research is used in various paragraphs thus maybe define this or use sustainability research and SE as unique terms. 

 Methods, Results- Figure two was not needed and is unclear to the reader. The narrative may  be adequate, Please indicate what steps were used for the development of the themes listed in the results. Please Add percentages to the quantitative data in the tables and figures. Maybe reduce quotes that are similar within the themes. 

 Conclusion - maybe add recommendations for pandemic preparedness beyond COVID -19 like vaccines

Is the work clearly and accurately presented and does it cite the current literature?

Yes

If applicable, is the statistical analysis and its interpretation appropriate?

I cannot comment. A qualified statistician is required.

Are all the source data underlying the results available to ensure full reproducibility?

Yes

Is the study design appropriate and is the work technically sound?

Yes

Are the conclusions drawn adequately supported by the results?

Partly

Are sufficient details of methods and analysis provided to allow replication by others?

Partly

Reviewer Expertise:

Social science research, ethics and stakeholder engagement within HIV prevention biomedical research.

I confirm that I have read this submission and believe that I have an appropriate level of expertise to confirm that it is of an acceptable scientific standard, however I have significant reservations, as outlined above.

# Reviewer report: Reviewer response for version 1

**DOI:** 10.5256/f1000research.159156.r308355

**Published:** 2024-07-31

**Authors:** Paul Upham

**Affiliations:** 1 University of Groningen, Groningen, The Netherlands

This is - in my view - a publishable-as-is paper that achieves its stated objectives of investigating and discussing how covid-19 affected stakeholder engagement in the context of sustainability research. Although simple in the sense of being primarily descriptive, it is clear that a lot of work went in to data gathering and analysis, the methods of which are all sound.

 Ideally the paper might reflect more on the implications of greater reliance on  online work for the humanities as well as the social sciences in sustainability research - for example, I wonder how online communication affects (positively and negatively) understanding in a phenomenological sense (if one can't 'be' with a stakeholder in their lived context). This could be briefly followed up in relation to an interviewee's comment on the challenges of interviewing fisher(men). Nonetheless the paper is useful and clear as it is.

Is the work clearly and accurately presented and does it cite the current literature?

Yes

If applicable, is the statistical analysis and its interpretation appropriate?

Yes

Are all the source data underlying the results available to ensure full reproducibility?

No source data required

Is the study design appropriate and is the work technically sound?

Yes

Are the conclusions drawn adequately supported by the results?

Yes

Are sufficient details of methods and analysis provided to allow replication by others?

Yes

Reviewer Expertise:

Social acceptance of energy technology; sociotechnical transitions studies; innovation studies

I confirm that I have read this submission and believe that I have an appropriate level of expertise to confirm that it is of an acceptable scientific standard.
